# Effect of Temporal and Spatial Smoothing on Speckle–Tracking-Derived Strain in Neonates

**DOI:** 10.1007/s00246-020-02536-3

**Published:** 2021-01-25

**Authors:** Umael Khan, Tom R. Omdal, Gottfried Greve, Ketil Grong, Knut Matre

**Affiliations:** 1 Department of Clinical Science, University of Bergen, Jonas Lies veg 87, 5021 Bergen, NO Norway; 2 Department of Heart Disease, Haukeland University Hospital, Bergen, Norway; 3 Department of Clinical Science, WestPaedResearch, University of Bergen, Bergen, Norway

**Keywords:** Speckle, Strain, Deformation, Neonatal, Smoothing, Myocardial

## Abstract

Clinical application of strain in neonates requires an understanding of which image acquisition and processing parameters affect strain values. Previous studies have examined frame rate, transmitting frequency, and vendor heterogeneity. However, there is a lack of human studies on how user-regulated spatial and temporal smoothing affect strain values in 36 neonates. This study examined nine different combinations of spatial and temporal smoothing on peak systolic left ventricular longitudinal strain in 36 healthy neonates. Strain values were acquired from four-chamber echocardiographic images in the software-defined epicardial, midwall, and endocardial layers in the six standard segments and average four-chamber stain. Strain values were compared using repeated measure ANOVAs. Overall, spatial smoothing had a larger impact than temporal smoothing, and segmental strain values were more sensitive to smoothing settings than average four-chamber strain. Apicoseptal strain decreased by approximately 4% with increasing spatial smoothing, corresponding to a 13–19% proportional change (depending on wall layer). Therefore, we recommend clinicians be mindful of smoothing settings when assessing segmental strain values.

**Supplementary Information:**

The online version contains supplementary material available at 10.1007/s00246-020-02536-3.

## Introduction

Left ventricular strain is a measure of left ventricular deformation [1]. When assessing left ventricular function in neonates, strain is regarded as a more sensitive measure of ventricular function than conventional echocardiographic measures such as shortening fraction (SF) and ejection fraction (EF) [2, 3], and is therefore gaining traction within neonatal cardiology. There are primarily two echocardiographic modalities for measuring strain, namely speckle-tracking echocardiography (STE) and tissue doppler imaging (TDI) [1]. In the clinical setting, STE is easier to use due to its relative angle independence and faster tracking compared to TDI and is a more widely applied imaging modality [1].

STE in neonates has shown great potential in assessment of various clinical conditions and is considered a more sensitive measure of ventricular function and dysfunction than conventional echocardiography [2]. Although STE could potentially be very useful within neonatal cardiology, obtaining strain measurements in neonates is particularly challenging due to small heart size and high heart rates [4]. For this reason, reference values in healthy neonates have begun to emerge in recent years and are still being established [2, 4, 5].

A range of factors including those related to image acquisition and image processing may affect STE strain values. Knowledge of how they affect strain values is necessary for proper clinical application of strain and to overcome the technical challenges of imaging in neonates. Improved quality of measurements could further enhance implementation of strain in a clinical setting. Previous studies in infants have primarily focused on the effect of vendor heterogeneity [6] or the role of user-regulated acquisition settings such as frame rate and transmitting frequency [5, 7]. This leaves an important knowledge gap regarding the effect of user-regulated image processing settings such as spatial and temporal smoothing. This is especially important as previous studies have indicated that the primary source of discordance in strain measurements is image processing rather than image acquisition [8]. Smoothing is an important element of image processing.

Both temporal and spatial smoothing are based on the application of cubic spline smoothing. Spline smoothing and interpolation is explained in more detail by Moen et al. and Pollock [9, 10]. Spatial smoothing implies that the strain measured in one part of the ventricle will be adjusted depending on strain measured in adjacent segments. Similarly, temporal smoothing means that one evens out the geometric transformations over time to achieve smoother transitions in strain between successive time intervals. The practical implications are that a high degree of smoothing results in smoother strain curves that are less subject to temporal fluctuations in strain values as well as a more uniform distribution of strain between segments. However, smoothing results in loss of resolution. In other words, by increasing smoothing, one risks losing strain data.

For neonatal cardiologists, it is important to know which acquisition parameters affect strain measurements and which do not in order to obtain reliable measures of strain. Unfortunately, there is a shortage of studies that assess how user-regulated spatial and temporal smoothing affect strain measurements. The purpose of this study was therefore to examine how strain values in healthy neonates are affected by spatial and temporal smoothing.

## Materials and Methods

Healthy neonates were enrolled into this study between June 2017 and June 2018. All neonates were recruited at Haukeland University Hospital, Bergen. We obtained written informed consent from parents as well as approval by The Regional Committee for Medical and Health Research Ethics (No. 2015/1918).

Inclusion criteria were healthy neonates born to term of mothers with no underlying pathology. A general echocardiographic examination of the neonates was done within the first month of life. Images were obtained from neonates in supine position in a calm, relaxed state. Images were acquired using a Vivid E9 scanner with a 12S pediatric cardiac probe with transmitted frequency 9 MHz (GE Vingmed Ultrasound, Horten, Norway). The frame rate was adjusted to obtain a frame rate/heart ratio > 1.0 [11]. Next, an image of the left ventricle was obtained in the apical four-chamber (4ch) view. Sector width and depth were adjusted so that only the left ventricle was visible in order to maximize beam density and frame rate [12].

The images were analyzed in the software EchoPAC v.202 (GE Vingmed Ultrasound, Horten, Norway). Timing of closure of the aortic valve was assessed through pulsed doppler measurements. Region of interest tracing was performed manually along the endocardial border of the left ventricle in accordance with the vendor guidelines. The thickness and placement of the region of interest was adjusted to cover the thickness of the ventricular wall while simultaneously avoiding inclusion of the pericardium. EchoPAC presents an assessment of the tracking quality. If any segments were deemed untraceable by EchoPAC or if the tracking was clearly faulty, the images were excluded.

**Fig. 1:**
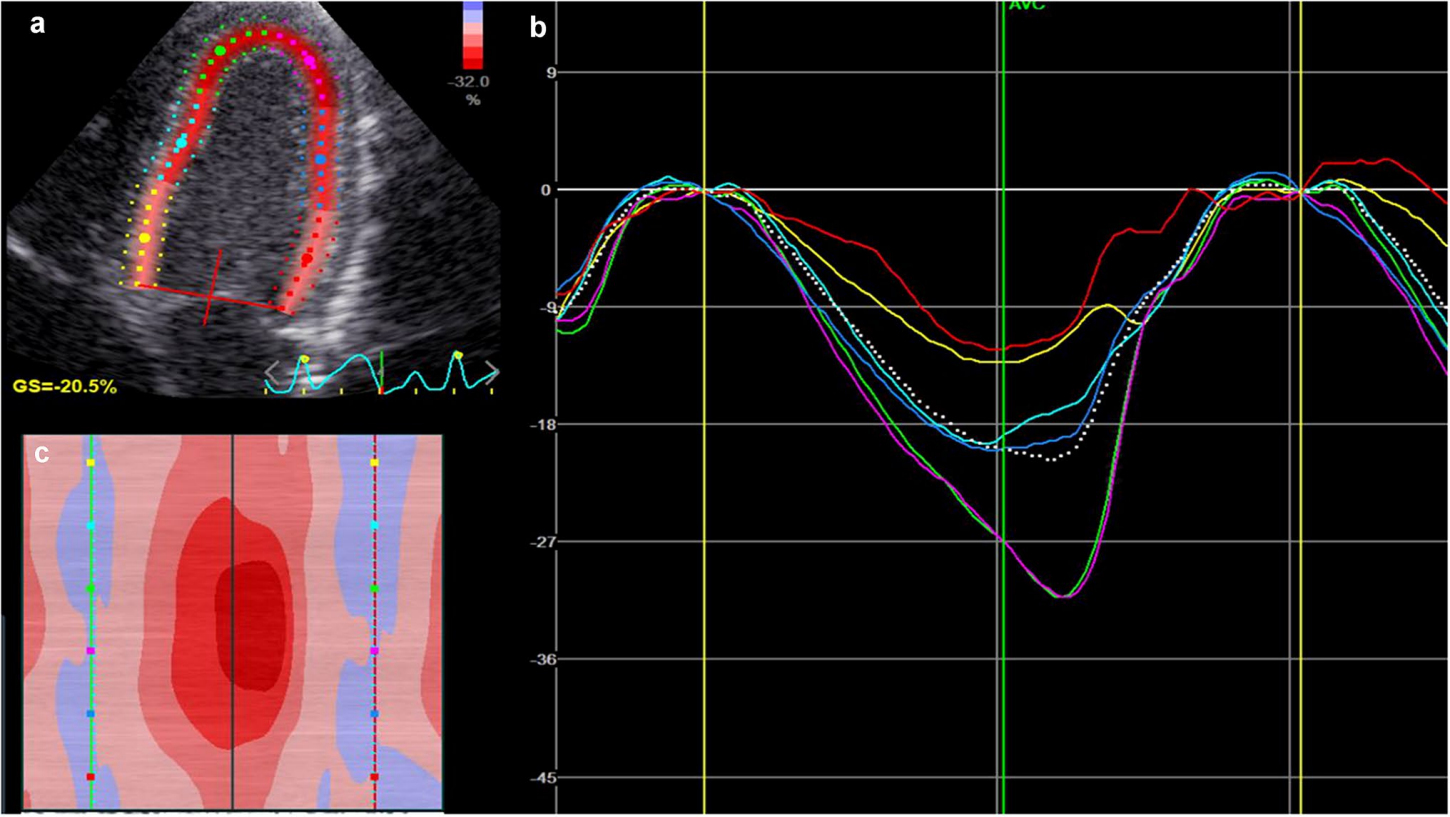
An example of a speckle-tracking echocardiography analysis. **a** The ultrasound image. **b** Six different curves with different colors, each corresponding to strain in a specific segment over time, in addition to the curve presenting average 4-chamber strain (broken curve). **c** Temporal changes in strain for each strain value, with high strain values represented as a darker red color

Peak systolic longitudinal strain was obtained for each of the six left ventricular segments in addition to peak averaged 4ch strain over the 6 segments in the three vendor-defined layers (endocardial, midwall, and epicardial). In order to assess the effect of smoothing, nine combinations of spatial smoothing and temporal smoothing were examined. These combinations are shown in Table 1. An example of strain curves is shown in Fig. 1. Thirty random analyses were selected for assessment of interobserver variability. Interobserver variability was assessed at the lowest, default and highest smoothing settings of both spatial and temporal smoothing (three combinations) for midwall strain, as this is the most commonly assessed layer.

**Table 1:** Examined smoothing settings

	Spatial smoothing			
		Low	Default	High
Temporal smoothing	Low	x*	x	x
	Default	x	x*	x
	High	x	x	x*

The nine examined combinations of smoothing settings, each marked with an x. The asterisks represent settings that were examined for interobserver reproducibility for midwall strain. Low refers to the lowest possible user-defined setting, default refers to the default setting for the software, which was medium, and high refers to the highest possible user-defined setting

### Statistical Approach

We used 21 two-way repeated measure ANOVAs with Bonferroni post-hoc multiple contrast tests to assess the effect of both temporal and spatial smoothing on strain measurements in the different segments as well as layers. The underlying assumption of sphericity was assessed using Mauchly’s test of sphericity, and Greenhouse-Geisser adjustment was applied when the assumption of sphericity was violated (*p* < 0.05). Normal distribution was assessed by the Shapiro–Wilk test of normality on the studentized residuals. The presence of outliers was assessed by examination of the studentized residuals greater than ± 3. Interobserver variability was assessed using a two-way mixed absolute agreement intraclass correlation coefficient. The analyses were carried out with the SPSS statistical package version 25 (SPSS Inc, IBM Corp., Armonk, NY, USA).

## Results

**Fig. 2:**
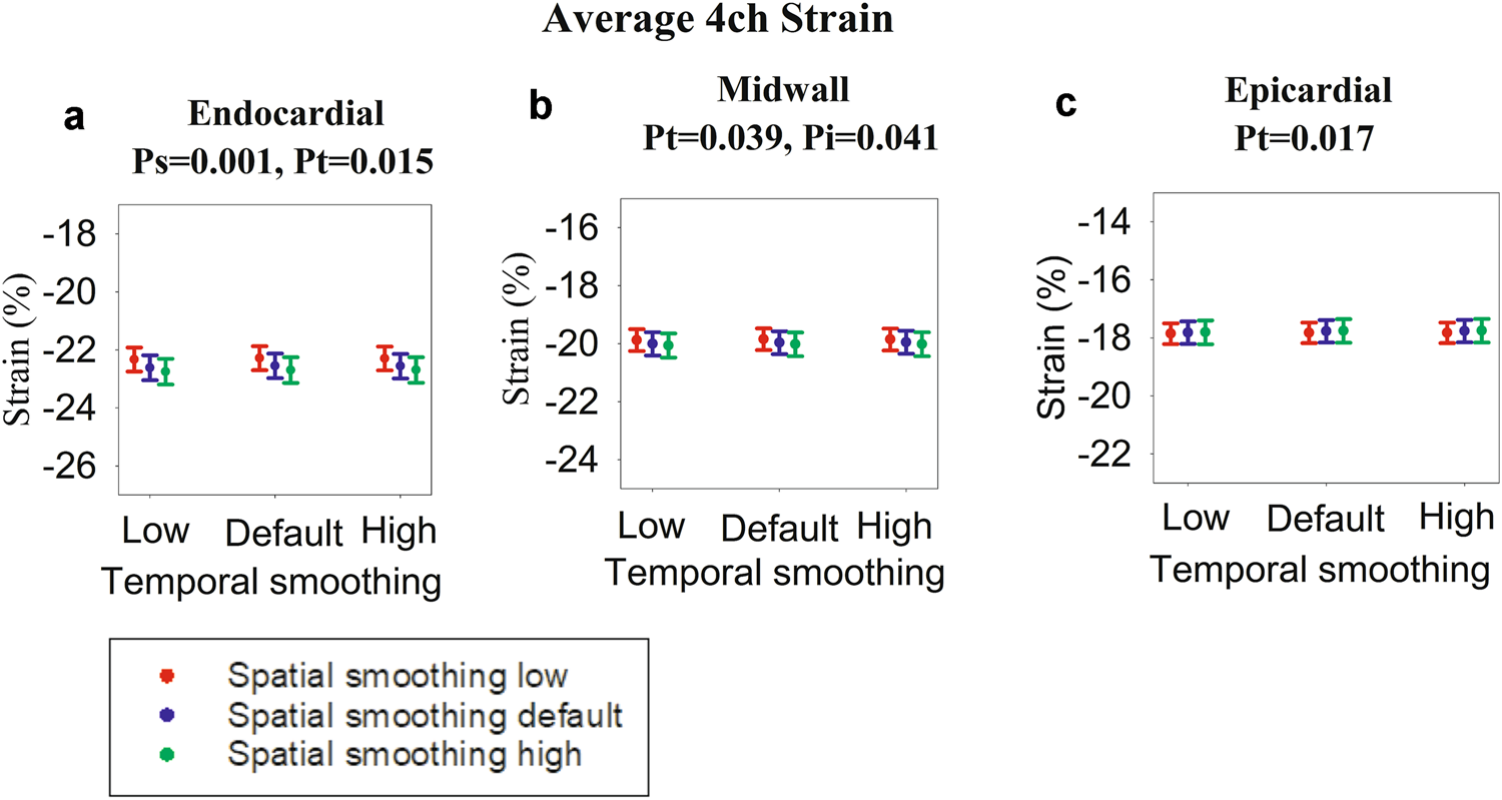
Average 4-chamber (4ch) strain. Each dot represents strain for a different setting of spatial and temporal smoothing. The error bars represent standard error of mean. For each graph, the significances (expressed as *p* values) of spatial smoothing (*p*_*i*_) and temporal smoothing (*p*_*t*_) were calculated, and in case of significance (*p* < 0.05), the *p* values are presented above the graphs. In addition, *p* values for interaction between spatial and temporal smoothing (*p*_*s*_) are presented if significant (*p* < 0.05). **a** Endocardial values, **b** Midwall values and **c** Epicardial values

**Fig. 3:**
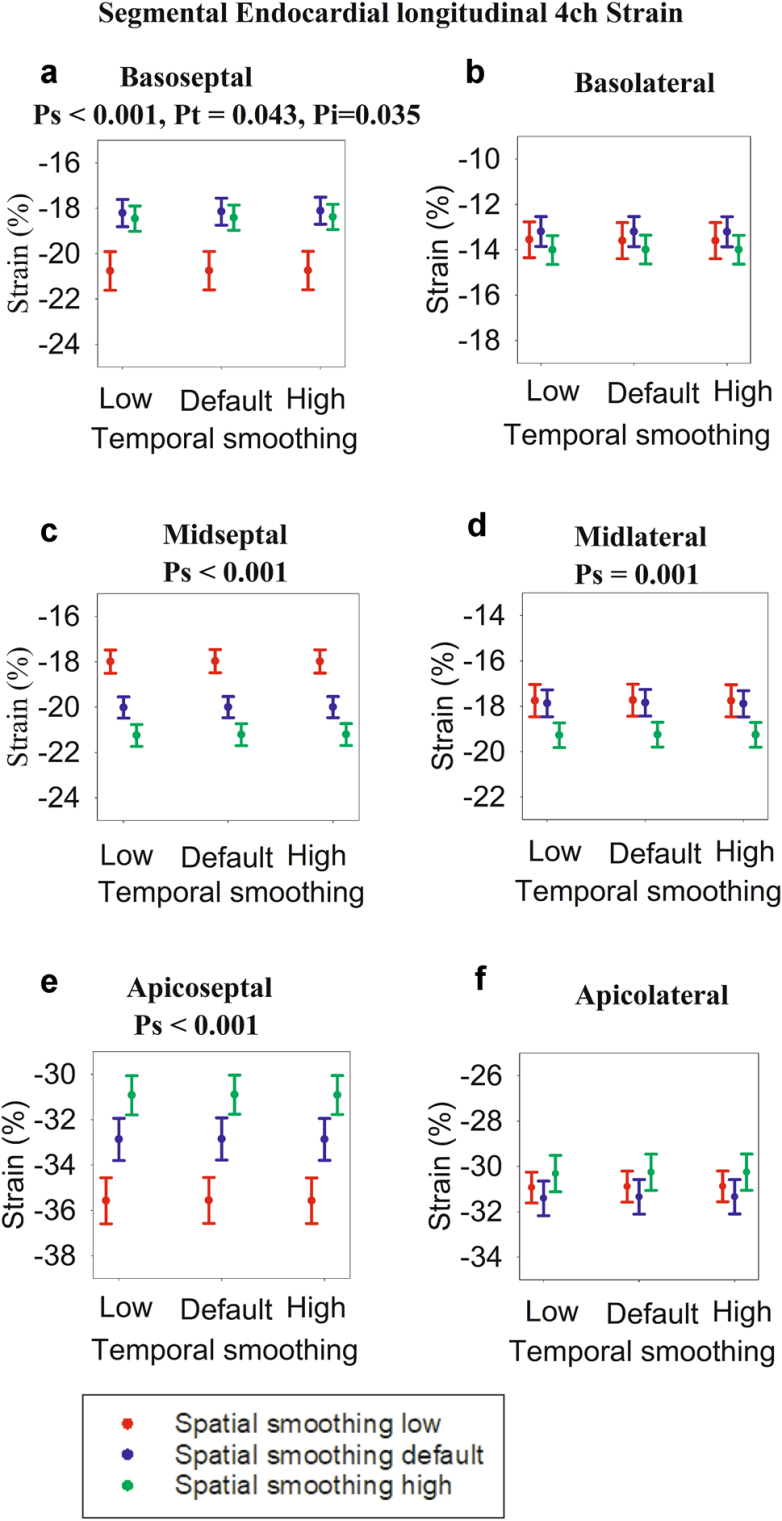
Endocardial segmental strain derived from 4-chamber view. Each dot represents strain for different settings of spatial and temporal smoothing. The error bars represent standard error of mean. For each graph, the significances (expressed as *p* values) of spatial smoothing (*p*_*s*_) and temporal smoothing (*p*_*t*_) were calculated, and in case of significance (*p* < 0.05), the *p* values are presented above the graphs. In addition, *p* values for interaction between spatial and temporal smoothing (*p*_*i*_) are presented if significance (*p* < 0.05). **a** Basoseptal segment, **b** Basolateral segment, **c** Midseptal segment, **d** Midlateral segment, **e** Apicoseptal segment, **f** Apicolateral segment

**Fig. 4:**
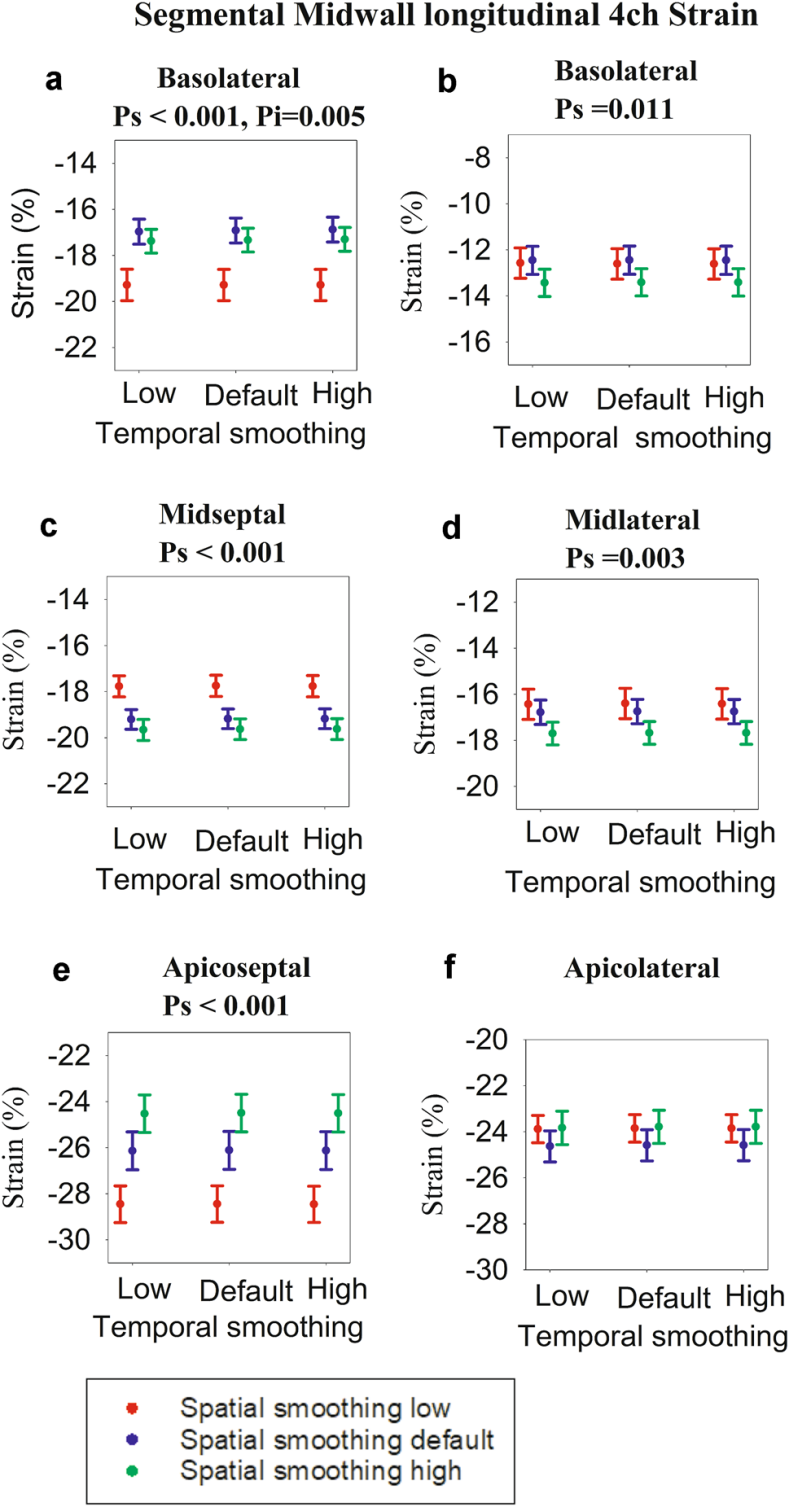
Midwall segmental strain derived from 4-chamber view. Each dot represents strain at a different setting for spatial and temporal smoothing. The error bars represent standard error of mean. For each graph, the significance (expressed as *p* values) of spatial smoothing (*p*_*s*_) and temporal smoothing (*p*_*t*_) were calculated, and in case of significance (*p* < 0.05), the *p* values are presented above the graphs. In addition, *p* values for interaction between spatial and temporal smoothing (*p*_*i*_) are presented if significance (*p* < 0.05). **a** Basoseptal segment, **b** Basolateral segment, **c** Midseptal segment, **d** Midlateral segment, **e** Apicoseptal segment, **f** Apicolateral segment

**Fig. 5:**
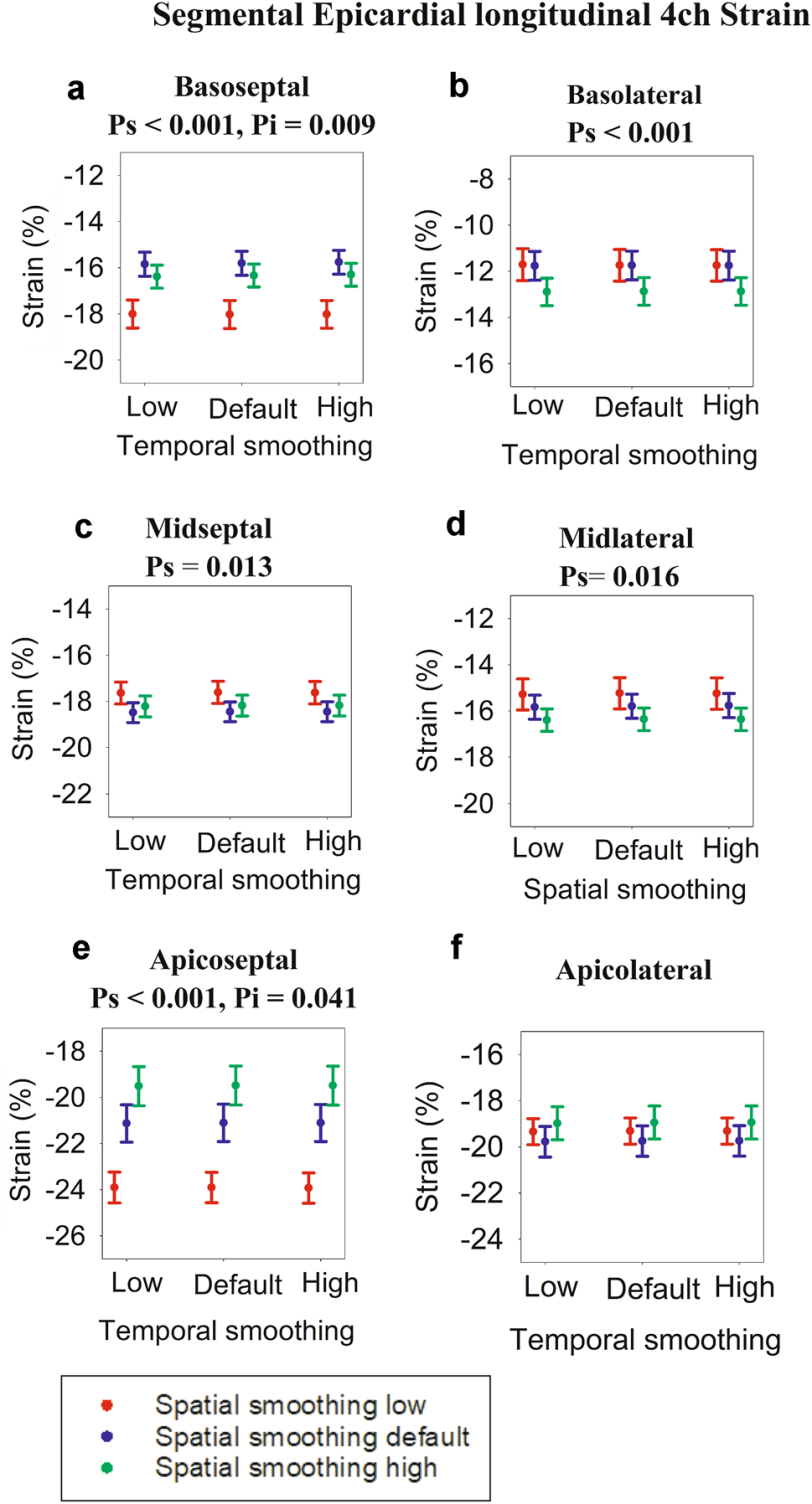
Epicardial segmental strain derived from 4-chamber view. Each dot represents strain at a different setting for spatial and temporal smoothing. The error bars represent standard error of mean. For each graph, the significance (expressed as *p* values) of spatial smoothing (*p*_*s*_) and temporal smoothing (*p*_*t*_) was calculated, and in case of significance (*p* < 0.05), the *p* values are presented above the graphs. In addition, *p* values for interaction between spatial and temporal smoothing (*p*_*i*_) are presented if significance (*p* < 0.05). **a** Basoseptal segment, **b** Basolateral segment, **c** Midseptal segment, **d** Midlateral segment, **e** Apicoseptal segment, **f** Apicolateral segment

Of the 39 neonates analyzed, three were excluded due to inadequate tracking of the basolateral segment, resulting in a total of 36 patients. The patient characteristics of the 36 included patients are shown in Table 2. The individual strain values for each setting are presented in Figs. 2, 3, 4, and 5, alongside the statistical significance for the overall effect of spatial and temporal smoothing. In addition, tables are provided in the electronic supplementary material that numerically present the strain values for every smoothing setting as well as the statistically significant mean differences in strain with changing smoothing settings. The statistically significant individual changes (*p* ≤ 0.05) from the post-hoc tests are described below. There was normal distribution in all data groups except in the apicolateral segments (Figs. 3f, 4f, and 5f, Shapiro–Wilk *p* value between 0.02 and 0.041 depending on the group) and the borderline case of the epicardial apicoseptal segment (Fig. 5e, Shapiro–Wilk *p* value between 0.047 and 0.048), corresponding to approximately 10% of the dataset. There are no equivalent well-established, non-parametric alternatives or transformations that were applicable to our dataset. There were single outliers in the apicolateral segments as well as the apicoseptal segment in the endocardial view and the midseptal and midlateral segments of the epicardial views. However, repeating the analyses without these outliers did not change the results.

**Table 2:** Patient characteristics and acquisition settings

Gender (number males/females)	20/16
Gestational age (weeks ± SD)	40 ± 1
Birth weight (grams ± SD)	3810 ± 569
Heart rate (BPM ± SD)	132 ± 22
Frame rate (FPS ± SD)	157 ± 24
Patent foramen ovale	67%
Patent ductus arteriosus	52%

*BPM* Beats per minute, *FPS* Frames per second, *SD* standard deviation

### Average 4ch Strain

The 4ch average strain values for each smoothing setting are displayed in the dot plots in Fig. 2, alongside the *p* values for the statistically significant smoothing settings. For endocardial 4ch average strain, strain increased for every incremental rise in spatial smoothing. At the same time, there was a reduction of strain when increasing temporal smoothing from low to default. The latter effect was also seen for epicardial 4ch strain. For midwall 4ch strain, increasing temporal smoothing from low to high reduced strain if spatial smoothing was set at default. The changes in strain, despite being statistically significant, were minimal with the greatest change being an increase of 0.4% in endocardial 4ch strain with increasing spatial smoothing, corresponding to a less than 2% proportional change in strain.

### Endocardial Segmental Strain

The individual endocardial segmental strain values for each smoothing setting are displayed in the dot plots in Fig. 3, alongside the *p* values for the statistically significant smoothing settings. In the endocardial basoseptal segment, increasing spatial smoothing from low to either default or high resulted in a decrease in strain. Increasing temporal smoothing resulted in decreased strain if spatial smoothing was either default or high. In the midseptal segment, strain increased with every increase of spatial smoothing, whereas it conversely decreased in the apicoseptal segment. The strain in the midlateral segment increased when spatial smoothing was increased from either default or low to high. The most pronounced change in strain was seen in the apicoseptal segment, with strain decreasing by 4.7% when strain spatial smoothing was increased from low to high. This corresponds to a 13.1% proportional change in strain.

### Midwall Segmental Strain

The individual midwall segmental strain values for each smoothing setting are displayed in the dot plots in Fig. 4, alongside the *p* values for the statistically significant smoothing settings. The overall trend is similar to what was seen for endocardial strain. In the midwall basoseptal segment, increasing spatial smoothing from low to default decreased strain, whereas further increasing spatial smoothing increased strain. Increasing temporal smoothing also reduced strain, provided spatial smoothing was default or high. In the midseptal, apicoseptal, and midlateral segments, the midwall displayed the same pattern as in the corresponding endocardial segments. In the basolateral region, increasing spatial smoothing from default to high led to an increase in strain.

### Epicardial Segmental Strain

The individual epicardial segmental strain values for each smoothing setting are displayed in the dot plots Fig. 5, alongside the *p* values for the statistically significant smoothing settings. In the basoseptal segment, changing smoothing showed a similar pattern as in the midwall layer of the basoseptal segment. In the midseptal segment, increasing spatial smoothing from low to default increased strain. The epicardial apicoseptal strain showed the same trend as the midwall layer of the epicardial segment, in addition to a slight decrease in strain when temporal smoothing was increased from low to default provided spatial smoothing was high. In the midlateral and basolateral segments, increasing spatial smoothing from low or default to high increased strain. Similar to the endocardial and midwall layers, the maximal change observed was the decrease in strain with increasing spatial smoothing, with the proportional change being 18.5%.

Table 3 demonstrates interobserver reliability at different levels of smoothing. Applying the definitions of Cicchetti et al. for evaluating intraclass correlation coefficient (below 0.40 is “poor”, 0.40–0.59 is “fair”, 0.60–0.74 is “good”, and above 0.75 is “excellent”) [13], the reliability was good to excellent for most settings, except in the basolateral segment when a combination of low spatial and temporal smoothing was applied.

**Table 3:** Interobserver variability for midwall strain

	Intraclass correlation coefficient		
	Low	Default	High
4-chamber average	0.964	0.911	0.896
Basoseptal	0.933	0.908	0.933
Midseptal	0.981	0.969	0.944
Apicoseptal	0.891	0.805	0.677
Apicolateral	0.833	0.890	0.814
Midlateral	0.916	0.896	0.895
Basolateral	0.221	0.674	0.826

Intraclass correlation coefficients for midwall strain at low, default, and high levels of spatial and temporal smoothing

## Discussion

The primary finding of this study was that segmental longitudinal strain values were more sensitive to smoothing settings than 4ch average strain. Although statistically significant effects for smoothing settings were seen for the 4ch average strain values, the maximal difference observed was in the range of 0.4% across smoothing settings, corresponding to 2% proportional change. Segmental values on the other hand, especially the apicoseptal segment, were more sensitive to spatial smoothing settings than average 4ch strain. The apicoseptal strain decreased by approximately 4% when spatial smoothing was increased from low to high, corresponding to approximately a 15% proportional change, depending on the layer being assessed. Currently, cut-off values for STE strain have yet to be established in neonates given the relative novelty of the method. In addition, segmental strain measures are less well established than GLS measures. With that caveat in mind, studies in older subjects show that a reduction in strain of similar magnitude is correlated with an increased risk of adverse cardiovascular events [14, 15]. Overall, spatial smoothing had a more pronounced effect than temporal smoothing. Finally, the septal segments displayed higher strain values and more sensitivity to smoothing than the lateral segments. This trend was seen in all three wall layers.

A previous animal study conducted on seven pericardiotomized pigs found no statistically significant effects of spatial and temporal smoothing on longitudinal strain, which corresponds well with the small changes seen in the average 4ch strain found in this study [9]. However, the animal study was conducted in an open chest, pericardiotomized setting and used a different segmentation model during its analysis. Therefore, a direct comparison between our results would be problematic.

Why were the segmental strain values more sensitive to smoothing than the average 4ch strain? When smoothing is applied, strain data are homogenized either across segments or across successive frames. When spatial smoothing is applied, one segment of the ventricle is partly adjusted according to strain in adjacent segments of the ventricle. However, the 4ch average strain is already the average of these segments. Thus, the inter-segment adjustment that takes place during smoothing would have a smaller effect on the average strain value across the segments than in individual segments.

The segment most sensitive to smoothing was the apicoseptal segment, where values decreased in conjunction with the increase in spatial smoothing. Figures 3, 4, and 5 demonstrate that strain increased from base to apex, and septal wall strain values were higher than lateral wall strain values. The apicoseptal segment thus had the highest average strain values. With increasing spatial smoothing, the neighboring as well as more basal segments likely reduced the apicoseptal strain values; hence the relative sensitivity of apicoseptal strain to spatial smoothing. Segments with low strain values find their strain values increased with increasing spatial smoothing, while the opposite takes place for segments high strain values. This implies that the degree of smoothing sensitivity is dependent on inter-segment strain heterogeneity and base to apex gradient in strain. Meta-analyses have revealed that this varies across studies, although the general trend is a base to apex gradient of strain [5, 16].

Temporal smoothing did not seem to affect strain as much as spatial smoothing. However, as temporal smoothing involves smoothing over successive frames, one could expect it to play a greater role at higher heart rates. Our images were obtained with a frame rate/heart rate ratio above 1 in accordance to recommendations from previous studies in neonates [7, 11]. The interaction between frame rate and temporal smoothing has not been sufficiently investigated. For instance, one could speculate that high frame rate could potentially have reduced the effect of increasing temporal smoothing. In future studies, it would be interesting to examine the effect of temporal smoothing at different frame rates. Our current recommendation is that temporal smoothing should be kept low, especially at high heart rates.

The lateral segments, especially the basolateral segment, seem to be challenging. Three patients were excluded from the present study based on poor tracking in the basolateral segment. This corresponds well with the poor interobserver reliability in the basolateral segment. The poor tracking quality could be a contributing factor in the reduced significance of smoothing in the lateral wall compared to the septal wall. Previous studies have reported difficult tracking of the lateral wall in adults and found more reproducible results in the septal wall compared to the other parts of the heart [8, 17]. Possible explanations included a more uniform acoustic field in the septal segments, less interference from the lung, and a motion through the cardiac cycle that is more parallel to the ultrasound beams compared to the lateral wall [8, 17]. In the apical views, the beam density decreases with distance from the apex, which could render tracking of the basolateral segment especially difficult.

Our study contributes to the ongoing effort to standardize and optimize speckle-tracking-derived strain measurements [18]. One can think of this effort as having two fronts: image acquisition and image processing. Sources of variability in image acquisition includes using different scanners [19], probes [11], transmitting frequency settings [20], and frame rate settings [7]. Sources of variability in image processing include use of different speckle-tracking software [21] and user-regulated settings within the software as was the case for this study. The result of such studies gives clinicians guidelines as to which settings are important to bear in mind when conducting speckle-tracking analyses [2]. To the best of our knowledge, this is the first study to examine the effect of user-regulated spatial and temporal smoothing in a human population.

The term “global longitudinal strain” is often a source of confusion. This is the most commonly assessed strain parameter. Meta-analyses show that most pediatric studies derive global longitudinal strain by averaging the six segments in the apical 4ch view [5, 16, 22], corresponding to what we refer to as average 4ch strain in this present study. However, others recommend that the term global longitudinal strain should be used for measurements that also incorporate the three-chamber and two-chamber views [5, 16, 22, 23]. This is further complicated by the fact that studies and meta-analyses differ in whether these different approaches have clinical implications [22, 24]. In order to avoid confusion, we have used the term average 4ch strain rather than global longitudinal strain in this study.

Clinical application of segmental strain is hampered due to suboptimal reproducibility [17]. This study indicates that some of the variability might stem from differences in smoothing. Interestingly, we see that increasing smoothing does not have a uniform effect on the intraclass correlation coefficient of the various segments, indicating that finding optimal levels of smoothing is not a simple task. While increasing smoothing improved the reproducibility of strain measurements in the basolateral segment, this was not seen in the other segments. In addition, one could speculate that simply increasing smoothing could lead to misinterpretation of regional pathologies, thereby decreasing the clinical sensitivity of speckle-tracking assessment of regional systolic cardiac function. Hence, we recommend keeping spatial smoothing at a minimum. This might be especially important when assessing segmental values of strain and regional pathologies. In conditions that reduce cardiac function in a more uniform fashion and when average strain is assessed rather than regional strain, higher levels of smoothing are acceptable. Also, longitudinal data obtained in the same patient at different smoothing settings are more acceptable for average strain values than regional strain values.

Multi-layer strain remains a controversial issue. Although it has not been extensively investigated in neonates, several studies have shown its utility in older subjects, for instance in the case of aortic stenosis [25], myocardial toxicity [26], and ischemic cardiomyopathy [27, 28]. Others argue that layer-specific strain might not be assessing independent layers at all, and any gradient seen is simply due to the incompressibility of myocardium [29, 30]. In the thin walls of the neonatal myocardium, one could further question how well STE actually is able to delineate and differentiate between the layers of the myocardial wall. Our study shows an endocardial to epicardial gradient in strain similar to what is seen in older subjects. As epicardial strain values are the lowest, it seems as if smoothing changes have the greatest impact in this layer. We recommend further studies into the clinical utility and variability in strain.

## Limitations

This study only examined longitudinal strain derived from the 4ch view, as this is the most basic and commonly assessed strain parameter. A complete assessment of GLS would also include the apical two-chamber and three-chamber views. Furthermore, it only assessed the effect of smoothing within the range that was provided to the user within EchoPAC software (GE Vingmed Ultrasound, Horten, Norway). This software was chosen as previous meta-analyses have shown it to be the most commonly used STE software [5, 16]. The findings of this study are therefore not directly applicable to other vendors or other strain directions. The goal of this study was simply to assess whether strain was affected by smoothing at all. It should also be noted that when assessing the role of any user-controlled setting, whether it can be smoothing or frame rate, one is only able to assess the effects of this setting within the range offered by the manufacturer. This study only examined neonates with smaller hearts and higher heart rates than what is seen in the adult population. Therefore, the results should be interpreted with caution when dealing with older patients. Finally, this study did not assess which smoothing settings are the most accurate. This would require a gold standard comparison method such as cardiac magnetic resonance imaging, which would be difficult in this age group. A comparison with implantable sensors like sonomicrometry is limited to in vitro and animal studies.

Unlike one-way repeated measure ANOVAs, which have non-parametric alternatives such as Friedman’s test, two-way repeated measure ANOVAs do not have well-established non-parametric alternatives. In our study, the non-normal data were in the apicolateral segments presented in Figs. 3f, 4f, and 5f and to a lesser extent the epicardial apicoseptal segment (Fig. 5e). If we exclude these, the overall trend and findings of this study nevertheless remain the same. On the other hand, ANOVAs are considered to be robust to deviations from normality [31]. Hence, ANOVAs for these segments were performed, but we recommend refraining from drawing absolute conclusions from this study for these particular segments.

## Conclusions

Average 4ch strain was quite robust with regard to smoothing settings. Conversely, segmental strain values were more sensitive to smoothing settings, especially in the apicoseptal segment. Spatial smoothing seems to play a more pronounced role than temporal smoothing for strain values. We recommend that the degree of smoothing be reported when presenting strain values, especially segmental strain values.

## Supplementary Information

Below is the link to the electronic supplementary material.

Supplementary file1 (PDF 191 KB)

## Data Availability

Data available on reasonable request from authors.
