# Supplementary material for: Effect of Temporal and Spatial Smoothing on Speckle–Tracking-Derived Strain in Neonates
Source: Pediatr Cardiol. 2021 Jan 25;42(4):743–52. doi: 10.1007/s00246-020-02536-3 (PMC8110490; doi:10.1007/s00246-020-02536-3)
Supplement: Supplementary file 1 — Supplementary file1 (PDF 191 KB) [file 246_2020_2536_MOESM1_ESM.pdf]

Effect of Temporal and Spatial Smoothing on Speckle Tracking Derived Strain in Neonates

Umael Khan[*], Tom R. Omdal[† ‡], Gottfried Greve[† ‡], Ketil Grong[*], Knut Matre[*]

[*] Department of Clinical Science, University of Bergen, Bergen, Norway

[†] Department of Heart Disease, Haukeland University Hospital, Bergen, Norway

[‡] WestPaedResearch, Department of Clinical Science, University of Bergen, Bergen, Norway

Corresponding author: Umael Khan, Department of Clinical Science, Jonas Lies veg 87, NO 5021 Bergen, Norway; telephone +4797521090; E-mail umael@hotmail.com

Supplementary table 1: Effect of smoothing on 4 chamber average longitudinal strain

Layer	Complete set of strain values at all combinations of spatial and temporal smoothing (%)				P values for overall effects of smoothing ($p \leq 0.05$ highlighted in bold type)	Statistically significant mean changes in strain (%) with changing spatial and temporal smoothing settings, alongside 95% confidence intervals of these mean differences. Non-significant changes not included.
Endocardial		**TL**	**TD**	**TH**	p_i=0.304<br>**p_s=0.001**<br>**p_t=0.015**	Spatial smoothing (no interaction with temporal smoothing):<br>SL-SD: 0.3 (95% CI 0.1 to 0.5)<br>SL-SH: 0.4 (95% CI 0.1 to 0.7)<br><br>Temporal smoothing (average across spatial smoothing):<br>TL-TD: 0.1 (95% CI 0.0 to 0.1)
	**SL**	-22.3±2.5	-22.3±2.5	-22.3±2.5		
	**SD**	-22.6±2.6	-22.5±2.5	-22.6±2.5		
	**SH**	-22.7±2.7	-22.7±2.7	-22.7±2.6		
Midwall		**TL**	**TD**	**TH**	**p_i=0.041**<br>p_s=0.168<br>**p_t=0.039**	Temporal smoothing (provided spatial smoothing set at default)<br>TL-TH: 0.1 (95% CI 0.0 to 0.1)
	**SL**	-19.9±2.3	-19.8±2.2	-19.9±2.2		
	**SD**	-20.0±2.4	-20.0±2.4	-19.9±2.4		
	**SH**	-20.1±2.5	-20.0±2.5	-20.0±2.5		
Epicardial		**TL**	**TD**	**TH**	p_i=0.113<br>p_s=0.680<br>**p_t=0.017**	Temporal smoothing (no interaction with spatial smoothing)<br>TL-TD*
	**SL**	-17.9±2.1	-17.8±2.1	-17.8±2.1		
	**SD**	-17.8±2.3	-17.8±2.3	-17.8±2.3		
	**SH**	-17.8±2.5	-17.8±2.4	-17.8±2.4		

CI: Confidence Interval; p_i: p-value for interaction between spatial and temporal smoothing; p_s: p-value for spatial smoothing; p_t: p-value for temporal smoothing; TD: Temporal smoothing default; TH: Temporal smoothing high; TL: Temporal smoothing low; SD: Spatial smoothing default; SH: Spatial smoothing high; SL: Spatial smoothing low *The difference between the segments was less than 0.1%, yet still statistically significant.

Supplementary table 2: Effect of smoothing on endocardial longitudinal strain

<table>
<tr><th>Segment</th><th colspan="4">Complete set of strain values at all combinations of spatial and temporal smoothing (%)</th><th>P values for overall effects of smoothing ($p \leq 0.05$ highlighted in bold type)</th><th>Statistically significant mean changes in strain (%) with changing spatial and temporal smoothing settings. alongside 95% confidence intervals of these mean differences. Non-significant changes not included.</th></tr>
<tr><td rowspan="4">Basoseptal</td><td></td><td>TL</td><td>TD</td><td>TH</td><td rowspan="4">p_s=0.001
p_t=0.043
p_i=0.035</td><td rowspan="4">Spatial smoothing (across all temporal smoothing setting):
SL-SD:-2.6 (95%CI: -1.5 to 3.7)
SL-SH:-2.3(95%CI: -1.1-3.6)
Temporal smoothing (provided spatial smoothing set at default):
TL-TD:-0.1(95%CI -0.1 to 0.0)
TL-TH:-0.1(95%CI -0.2 to 0.0)
TD-TH*
Temporal smoothing (provided spatial smoothing set at high):
TL-TD*
TL-TH:-0.1(95%CI -0.1 to 0.0)
TD-TH*</td></tr>
<tr><td>SL</td><td>-20.8±5.1</td><td>-20.7±5.1</td><td>-20.7±5.1</td></tr>
<tr><td>SD</td><td>-18.2±3.6</td><td>-18.1±3.6</td><td>-18.1±3.6</td></tr>
<tr><td>SH</td><td>-18.5±3.4</td><td>-18.4±3.3</td><td>-18.4±3.3</td></tr>
<tr><td rowspan="4">Midseptal</td><td></td><td>TL</td><td>TD</td><td>TH</td><td rowspan="4">p_s<0.001
p_t=0.342
p_i=0.279</td><td rowspan="4">Spatial smoothing (across all temporal smoothing setting):
SL-SD:2.0 (95%CI:1.3 to 2.8)
SL-SH:3.2(95%CI: 2.2 to 4.2)
SD-SH:1.2(95%CI: 0.9 to 1.5)</td></tr>
<tr><td>SL</td><td>-18.0±3.1</td><td>-18.0±3.1</td><td>-18.0±3.1</td></tr>
<tr><td>SD</td><td>-20.0±2.8</td><td>-20.0±2.8</td><td>-20.0±2.8</td></tr>
<tr><td>SH</td><td>-21.2±2.9</td><td>-21.2±2.9</td><td>-21.2±2.9</td></tr>
<tr><td rowspan="4">Apicoseptal</td><td></td><td>TL</td><td>TD</td><td>TH</td><td rowspan="4">p_s<0.001
p_t=0.460
p_i=0.892</td><td rowspan="4">Spatial smoothing (across all temporal smoothing setting):
SL-SD:-2.7 (95%CI: -3.4 to -2.0)
SL-SH:-4.7 (95%CI: -5.7 to -3.6)
SD-SH:-2.0 (95%CI: -1.5 to -2.4)</td></tr>
<tr><td>SL</td><td>-35.6±6.1</td><td>-35.6±6.1</td><td>-35.6±6.1</td></tr>
<tr><td>SD</td><td>-32.9±5.6</td><td>-32.9±5.6</td><td>-32.9±5.6</td></tr>
<tr><td>SH</td><td>-30.9±5.2</td><td>-30.9±5.2</td><td>-30.9±5.2</td></tr>
<tr><td rowspan="4">Apicolateral</td><td></td><td>TL</td><td>TD</td><td>TH</td><td rowspan="4">p_s=0.060
p_t=0.070
p_i=0.800</td><td rowspan="4"></td></tr>
<tr><td>SL</td><td>-30.9±4.1</td><td>-30.9±4.1</td><td>-30.9±4.1</td></tr>
<tr><td>SD</td><td>-31.4±4.6</td><td>-31.3±4.6</td><td>-31.3±4.6</td></tr>
<tr><td>SH</td><td>-30.3±4.8</td><td>-30.3±4.8</td><td>-30.3±4.8</td></tr>
<tr><td rowspan="4">Midlateral</td><td></td><td>TL</td><td>TD</td><td>TH</td><td rowspan="4">p_s=0.001
p_t=0.441
p_i=0.479</td><td rowspan="4">Spatial smoothing (across all temporal smoothing setting):
SL-SH:1.5 (95%CI: 0.4 to 2.6)
SD-SH:1.4 (95%CI: 0.9 to 1.9)</td></tr>
<tr><td>SL</td><td>-17.8±4.3</td><td>-17.7±4.2</td><td>-17.8±4.2</td></tr>
<tr><td>SD</td><td>-17.9±3.5</td><td>-17.8±3.5</td><td>-17.9±3.5</td></tr>
<tr><td>SH</td><td>-19.3±3.3</td><td>-19.3±3.3</td><td>-19.3±3.3</td></tr>
<tr><td rowspan="3">Basolateral</td><td></td><td>TL</td><td>TD</td><td>TH</td><td rowspan="3">p_s=0.135
p_t=0.829
p_i=0.425</td><td rowspan="3"></td></tr>
<tr><td>SL</td><td>-13.6±4.7</td><td>-13.6±4.8</td><td>-13.6±4.8</td></tr>
<tr><td>SD</td><td>-13.2±4.0</td><td>-13.2±4.0</td><td>-13.2±4.0</td></tr>
</table>

	**SH**	-14.0±3.8	-14.0±3.8	-14.0±3.8		

CI: Confidence Interval; p_i: p-value for interaction between spatial and temporal smoothing; p_s: p-value for spatial smoothing; p_t: p-value for temporal smoothing; TD: Temporal smoothing default; TH: Temporal smoothing high; TL: Temporal smoothing low; SD: Spatial smoothing default; SH: Spatial smoothing high; SL: Spatial smoothing low

*The difference between the segments was less than 0.1%. yet still statistically significant.

Supplementary table 3: Effect of smoothing on midwall longitudinal strain

Segment	Complete set of strain values at all combinations of spatial and temporal smoothing (%)				P values for overall effects of smoothing ($p \leq 0.05$ highlighted in bold type)	Statistically significant mean changes in strain (%) with changing spatial and temporal smoothing settings. alongside 95% confidence intervals of these mean differences. Non-significant changes not included.
Basoseptal		**TL**	**TD**	**TH**	**p_s<0.001**<br>p_t=0.068<br>**p_i=0.005**	Spatial smoothing (temporal smoothing low)<br>SL-SD:-2.4(95%CI:-3.2 to -1.6)<br>SL-SH:-1.9(95%CI:-2.8 to -1.0)<br>SD-SH: 0.4(95%CI: -0.2 to -0.7)<br>Spatial smoothing (temporal smoothing default)<br>SL-SD:-2.4(95%CI:-3.2 to -1.6)<br>SL-SH:-1.9(95%CI:-2.8 to -1.0)<br>SD-SH: 0.4(95%CI: -0.2 to -0.7)<br>Spatial smoothing (temporal smoothing default)<br>SL-SD:-2.4(95%CI:-3.2 to -1.6)<br>SL-SH:-2.0(95%CI:-2.8 to -1.1)<br>SD-SH: 0.4(95%CI: -0.2 to -0.7)<br>Temporal smoothing (spatial smoothing default)<br>TL-TD:-0.1(95%CI:0.0 to -0.1)<br>TL-TH:-0.1(95%CI: 0.0 to -0.2)<br>TD-TH*<br>Temporal smoothing (spatial smoothing high)<br>TL-TD*<br>TL-TH:-0.1(95%CI: 0.0 to -0.1)<br>TD-TH*
	**SL**	-19.3±4.1	-19.3±4.1	-19.3±4.1		
	**SD**	-17.0±3.3	-16.9±3.3	-16.9±3.2		
	**SH**	-17.4±3.1	-17.3±3.1	-17.3±3.1		
Midseptal		**TL**	**TD**	**TH**	**p_s<0.001**<br>p_t=0.241<br>p_i=0.293	Spatial smoothing (across all temporal smoothing setting):<br>SL-SD: 1.4 (95%CI: 0.8 to 2.0)<br>SL-SH: 1.9 (95%CI:1.0 to 2.7)<br>SD-SH*
	**SL**	-17.8±2.7	-17.7±2.8	-17.8±2.8		
	**SD**	-19.2±2.6	-19.2±2.6	-19.2±2.6		
	**SH**	-19.7±2.7	-19.6±2.7	-19.6±2.7		
Apicoseptal		**TL**	**TD**	**TH**	**p_s<0.001**<br>p_t=0.373<br>p_i=0.279	Spatial smoothing (across all temporal smoothing setting):<br>SL-SD: -2.3 (95%CI:-3.0 to -1.7)<br>SL-SH: -3.9 (95%CI:-4.9 to -3.0)<br>SD-SH: -1.6 (95%CI:-1.9 to -1.6)
	**SL**	-28.5±4.8	-28.5±4.7	-28.5±4.7		
	**SD**	-26.1±5.0	-26.1±4.9	-26.1±4.9		
	**SH**	-24.5±4.9	-24.5±4.9	-24.5±4.9		
Apicolateral		**TL**	**TD**	**TH**	p_s=0.068<br>p_t=0.061<br>p_i=0.688	
	**SL**	-23.9±3.6	-23.9±3.6	-23.9±3.6		
	**SD**	-24.6±4.1	-24.6±4.0	-24.6±4.0		
	**SH**	-23.8±4.3	-23.8±4.3	-23.8±4.3		
Midlateral		**TL**	**TD**	**TH**		

	**SL**	-16.4±4.0	-16.4±4.0	-16.4±4.0	**p_s=0.003** p_t=0.196 p_i=0.685	Spatial smoothing (across all temporal smoothing setting): SL-SH: 1.3 (95%CI: 0.2 to 2.3) SD-SH: 0.9 (95%CI: 0.5 to 1.4)
	**SD**	-16.8±3.2	-16.7±3.2	-16.8±3.2		
	**SH**	-17.7±3.0	-17.7±3.0	-17.7±3.0		
Basolateral		**TL**	**TD**	**TH**	**p_s=0.011** p_t=0.935 p_i=0.365	Spatial smoothing (across all temporal smoothing setting): SD-SH: 1.0 (95%CI:0.6 to 1.4)
	**SL**	-12.6±4.0	-12.6±4.0	-12.6±4.0		
	**SD**	-12.5±3.7	-12.4±3.7	-12.4±3.7		
	**SH**	-13.4±3.5	-13.4±3.5	-13.4±3.5		

CI: Confidence Interval; p_i: p-value for interaction between spatial and temporal smoothing; p_s: p-value for spatial smoothing; p_t: p-value for temporal smoothing; TD: Temporal smoothing default; TH: Temporal smoothing high; TL: Temporal smoothing low; SD: Spatial smoothing default; SH: Spatial smoothing high; SL: Spatial smoothing low
*The difference between the segments was less than 0.1%. yet still statistically significant.

Supplementary table 4: Effect of smoothing on epicardial longitudinal strain

Segment	Complete set of strain values at all combinations of spatial and temporal smoothing (%)				P values for overall effects of smoothing (p≤0.05 highlighted in bold type)	Statistically significant mean changes in strain (%) with changing spatial and temporal smoothing settings. alongside 95% confidence intervals of these mean differences. Non-significant changes not included.
Basoseptal		**TL**	**TD**	**TH**	**p_s<0.001**<br>p_t=0.124<br>**p_i=0.009**	Spatial smoothing (temporal smoothing low)<br>SL-SD:-2.2(95%CI:-2.9 to -1.4)<br>SL-SH:-1.6(95%CI:-2.5 to -0.8)<br>SD-SH: 0.5(95%CI:0.8 to 0.3)<br>Spatial smoothing (temporal smoothing default)<br>SL-SD: -2.2(95%CI:-2.9 to -1.5)<br>SL-SH: -1.7(95%CI:-2.5 to -0.9)<br>SD-SH: 0.5(95%CI:0.8 to 0.3)<br>Spatial smoothing (temporal smoothing default)<br>SL-SD: -2.3(95%CI:-3.0 to -1.5<br>SL-SH: -1.7(95%CI:-2.5 to -0.9)<br>SD-SH: 0.5(95%CI:0.3 to 0.8)<br>Temporal smoothing (spatial smoothing default)<br>TL-TH:-0.1(95%CI:-0.2 to 0.0)<br>TD-TH*<br>Temporal smoothing (spatial smoothing high)<br>TL-TD:-0.1(95%CI:-0.1 to 0.0)<br>TL-TH:-0.1(95%CI:-0.1 to 0.0)<br>TD-TH*
	**SL**	-18.0±3.7	-18.0±3.6	-18.0±3.6		
	**ST**	-15.8±3.1	-15.8±3.1	-15.8±3.1		
	**SH**	-16.4±3.0	-16.3±3.0	-16.3±3.0		
Midseptal		**TL**	**TD**	**TH**	**p_s=0.013**<br>p_t=0.219<br>p_i=0.403	Spatial smoothing (across all temporal smoothing setting):<br>SL-SD: 0.8 (95%CI:0.3 to 1.4)
	**SL**	-17.6±2.9	-17.6±2.9	-17.6±2.9		
	**ST**	-18.5±2.6	-18.4±2.6	-18.4±2.6		
	**SH**	-18.2±2.7	-18.2±2.7	-18.2±2.7		
Apicoseptal		**TL**	**TD**	**TH**	**p_s<0.001**<br>p_t=0.413<br>**p_i=0.041**	Spatial smoothing (temporal smoothing low)<br>SL-SD:-2.8(95%CI:-3.7 to -1.9)<br>SL-SH:-4.4(95%CI:-5.6 to -3.2)<br>SD-SH: -1.6(95%CI:-1.9 to -1.3)<br>Spatial smoothing (temporal smoothing low)<br>SL-SD:-2.8(95%CI:-3.7 to -1.9)<br>SL-SH:-4.4(95%CI:-5.6 to -3.3)<br>SD-SH: -1.6(95%CI:-1.9 to -1.3)<br>Spatial smoothing (temporal smoothing low)<br>SL-SD:-2.8(95%CI:-3.7 to -1.9)<br>SL-SH:-4.4(95%CI:-5.6 to -3.3)<br>SD-SH: -1.6(95%CI:-1.9 to -1.3)<br>Spatial smoothing (temporal smoothing low)<br>SL-SD:-2.8(95%CI:-3.7 to -1.9)<br>SL-SH:-4.4(95%CI:-5.6 to -3.3)
	**SL**	-23.9±4.0	-23.9±4.0	-23.9±3.9		
	**ST**	-21.1±4.9	-21.1±4.9	-21.1±4.8		
	**SH**	-19.5±5.1	-19.5±5.1	-19.5±5.1		

						SD-SH: -1.6(95%CI:-1.9 to -1.3)<br>Temporal smoothing (spatial smoothing high)<br>TL-TD*
Apicolateral		**TL**	**TD**	**TH**	p_s=0.111<br>p_t=0.131<br>p_i=0.759	
	**SL**	-19.3±3.4	-19.3±3.4	-19.3±3.4		
	**ST**	-19.8±4.0	-19.7±4.0	-19.7±3.9		
	**SH**	-19.0±4.3	-18.9±4.3	-18.9±4.3		
Midlateral		**TL**	**TD**	**TH**	**p_s=0.016**<br>p_t=0.063<br>p_i=0.418	Spatial smoothing (across all temporal smoothing setting):<br>SL-SH: 1.1 (95%CI:0.0 to 2.2)<br>SD-SH: 0.6 (95%CI:0.1 to 1.1)
	**SL**	-15.3±4.0	-15.2±4.1	-15.2±4.1		
	**ST**	-15.8±3.1	-15.8±3.1	-15.8±3.1		
	**SH**	-16.4±2.9	-16.4±2.9	-16.4±2.9		
Basolateral		**TL**	**TD**	**TH**	**p_s=0.001**<br>p_t=0.957<br>p_i=0.374	Spatial smoothing (across all temporal smoothing setting):<br>SL-SH: 1.2 (95%CI:0.2 to 2.1)<br>SD-SH: 1.1 (95%CI:0.7 to 1.5)
	**SL**	-11.7±4.2	-11.7±4.1	-11.7±4.1		
	**ST**	-11.8±3.7	-11.7±3.7	-11.7±3.7		
	**SH**	-12.9±3.6	-12.9±3.6	-12.9±3.6		

p_i: p-value for interaction between spatial and temporal smoothing; p_s: p-value for spatial smoothing; p_t: p-value for temporal smoothing; TD: Temporal smoothing default; TH: Temporal smoothing high; TL: Temporal smoothing low; SD: Spatial smoothing default; SH: Spatial smoothing high; SL: Spatial smoothing low

*The difference between the segments was less than 0.1%. yet still statistically significant.
